# Long‐term prognosis of pure and impure tachycardiomyopathy

**DOI:** 10.1002/ehf2.15444

**Published:** 2025-10-09

**Authors:** Giulia Stronati, Michele Alfieri, Niki Tombolesi, Alessandro Barbarossa, Samuele Principi, Federico Gullì, Arianna Massari, Gianmarco Bastianoni, Francesca Roccetti, Michela Casella, Antonio Dello Russo, Federico Guerra

**Affiliations:** ^1^ Department of Biomedical Sciences and Public Health Marche Polytechnic University, Marche University Hospital Ancona Italy; ^2^ Cardiology and Intensive Care Unit IRCCS INRCA Ancona Italy; ^3^ Cardiology and Arrhythmology Clinic Marche University Hospital Ancona Italy; ^4^ Department of Odontostomatologic and Specialized Clinical Sciences Marche Polytechnic University, Marche University Hospital Ancona Italy

**Keywords:** arrhythmia‐induced cardiomyopathy, atrial fibrillation, heart failure, prognosis, survival, tachycardiomyopathy

## Abstract

**Background and aims:**

Tachycardia‐induced cardiomyopathy (TCM) is a reversible form of heart failure (HF) driven by arrhythmias, often atrial fibrillation (AF). While reversible, TCM's long‐term prognosis remains unclear, especially in comparison to HF with reduced ejection fraction (HFrEF). This study examines the prognosis of pure and impure TCM against other causes of HFrEF.

**Methods:**

Prospective, monocentric, observational study of 456 patients hospitalized with de novo, acute decompensated HFrEF, classified into pure TCM, impure TCM, ischaemic HF and non‐ischaemic HF. The primary endpoint was all‐cause mortality, and the secondary endpoint was the incidence of unplanned cardiovascular hospitalisations. Sensitivity analyses were performed using propensity score matching between the four groups.

**Results:**

During a median follow‐up of 3 years (interquartile range 1.5–5.1 years), pure TCM had the highest survival rate, and ischaemic HF had the lowest (pure TCM 78.2%; impure TCM 64.8%; non‐ischaemic HF 73.4%; ischaemic HF 58.5%; log‐rank *P* < 0.0001). Pure and impure TCM presented the lowest free‐from‐readmission estimates over follow‐up (pure TCM 43.2%; impure TCM 60.0%; non‐ischaemic HF 83.2%; ischaemic HF 69.9%; log‐rank *P* < 0.0001). An initial rhythm control strategy was associated with better overall survival in TCM (79% vs. 63%; log‐rank *P* < 0.0001) but similar rates of unplanned hospitalization.

**Conclusions:**

Pure TCM shows a favourable survival prognosis but high readmission rates, emphasizing the need for early rhythm control and sustained monitoring for arrhythmia recurrence. An initial rhythm control strategy seems associated with an increased survival, highlighting the importance of early recognition of arrhythmias as a culprit of HF worsening.

## Introduction

Tachycardia‐induced cardiomyopathy or tachycardiomyopathy (TCM) is an important cause of left ventricular dysfunction and heart failure (HF).^1^ Both atrial and ventricular arrhythmias, more frequently represented by atrial fibrillation (AF) or atrial flutter, can cause the impairment of left ventricular function.^1^, ^2^ TCM is considered to be partially or fully reversible once the underlying cause is resolved.^3^

Two variants of the condition have been described: pure TCM or arrhythmia‐induced tachycardiomyopathy, in which the arrhythmia is the sole reason for the dysfunction, and impure TCM or arrhythmia‐mediated TCM, where the arrhythmia can exacerbate or worsen HF or an underlying heart disease.^1^, ^4^, ^5^

The current prevalence of TCM is not yet defined, although it accounts for almost 10% of all hospitalizations for acute HF in patients with no underlying structural heart disease and tends to recur over time.^6^

There is currently no standardized treatment for TCM, although the main aim is an early recognition of the condition and subsequent implementation of strategies to either obtain rhythm control or rate control. One treatment strategy that is advancing is rhythm control by catheter ablation in patients with TCM, which is currently recommended by the new AF guidelines^7^ and seems to be characterized by a good post‐procedural outcome.^8^ Nonetheless, awareness of TCM as a potential cause of acute HF is critical, and little is known about the long‐term prognosis of such a condition, especially when compared with more ‘traditional’ forms of HF with reduced ejection fraction (HFrEF).

The present study aims to assess the prognosis of patients with different types of TCM (pure vs. impure) and compare them with patients with different subtypes of HFrEF.

## Methods

### Study population

The present paper is a prospective observational study enrolling consecutive patients admitted with a de novo acute decompensated HFrEF diagnosis in the Cardiology and Arrhythmology Clinic of the Marche University Hospital of Ancona, Italy, from January 2018 to January 2022. The Cardiology and Arrhythmology Clinic provides consultancy services related to cardiac diseases to the regional admission and emergency hub and is specialized in arrhythmia and HF acute management and treatment.

All patients were treated according to HF guidelines available at the time and followed up after discharge in our ambulatory clinic. The end of the follow‐up was January 2025. All patients were divided into four subgroups according to clinical characteristics: pure TCM, impure TCM, ischaemic HF and non‐ischaemic HF, each defined as follows:

- Pure TCM: LV dysfunction solely secondary to acute arrhythmias and no underlying heart disease. Evidence of recovery after arrhythmia treatment, defined as an improvement of at least one New York Heart Association (NYHA) class and the recovery of at least five points of the left ventricular ejection fraction (LVEF) during the follow‐up.^1^, ^9^, ^10^ Due to a specific lack of international consensus regarding TCM‐related improvements, post‐hoc sensitivity analyses were subsequently performed using stricter LVEF cut‐offs (see below).
- Ischaemic HF: de novo acute HF in patients with ischaemic heart disease and no evidence of arrhythmias as a potential culprit for the acute decompensation.
- Non‐ischaemic HF: de novo acute HF in patients who did not meet the definition of ischaemic HF despite a thorough investigation, and no evidence of arrhythmias as a potential culprit for the acute decompensation. This group included both idiopathic dilated and non‐dilated left ventricular cardiomyopathy phenotypes.
- Impure TCM: LV dysfunction mediated by an acute arrhythmia that exacerbated an underlying heart disease, as described above, and evidence of recovery after arrhythmia treatment.^10^

Patients with primary valvular heart disease, other types of cardiomyopathies, Takotsubo syndrome and congenital heart diseases were excluded, as were patients with inflammatory or infiltrative causes of HF. Similarly, patients with other clinical presentations, such as acute pulmonary oedema, isolated right ventricular failure, and cardiogenic shock were excluded.

**Figure 1:**
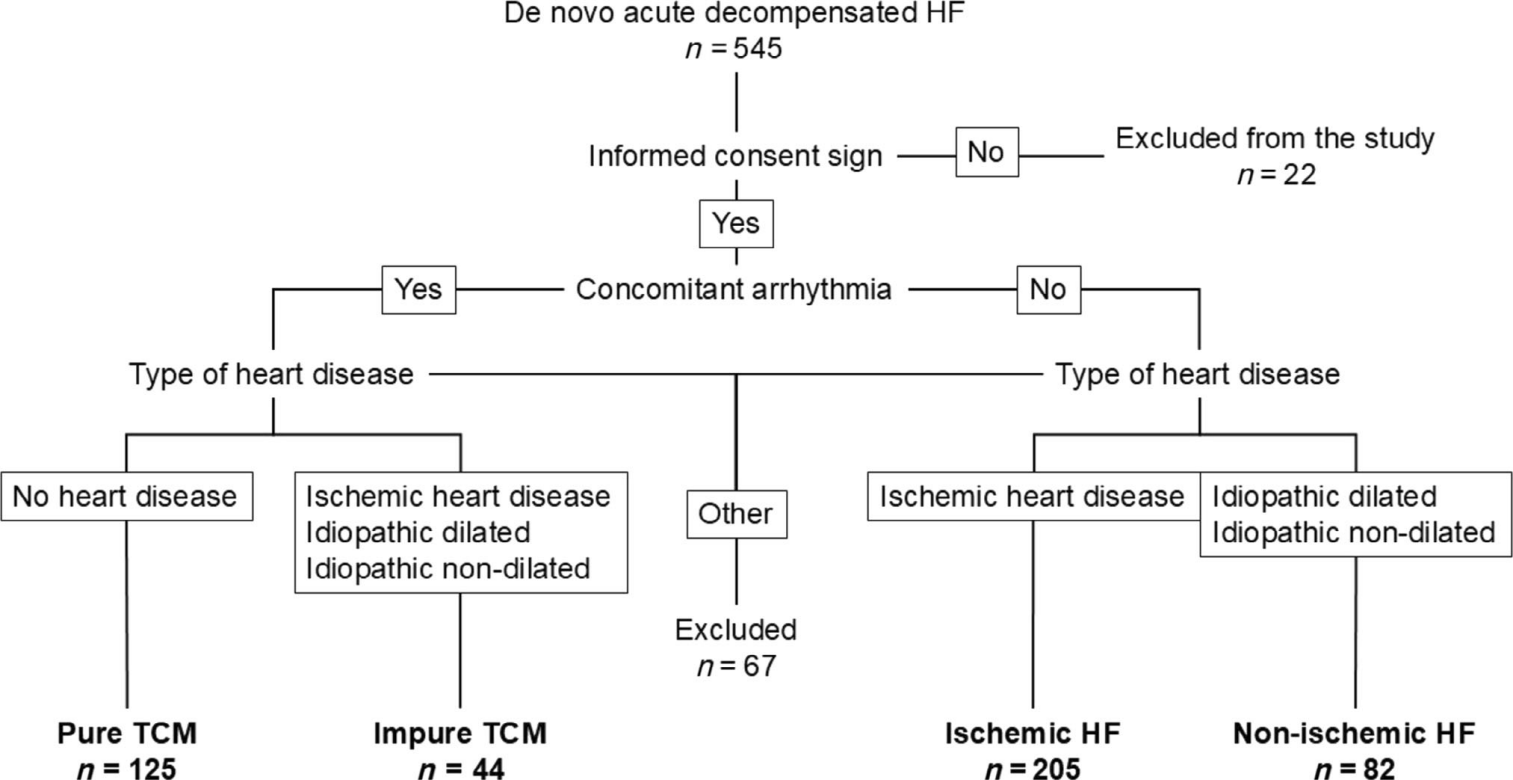
Diagnostic flowchart and subgroup allocation. HF, heart failure.

A summary of the diagnostic flowchart and subgroup allocation is presented in *Figure* 1.

Ischaemic heart disease was characterized by a history of revascularization or significant coronary obstruction observed during hospitalization via coronary angiography, as previously detailed.^6^ Valvular heart disease was defined by prior aortic or mitral replacement or repair, evidence of severe aortic or mitral regurgitation, significant aortic stenosis or moderate to severe mitral stenosis. To rigorously detect idiopathic dilated and non‐dilated left ventricular cardiomyopathy phenotypes, all patients underwent a comprehensive diagnostic evaluation. This included the collection of a detailed, multi‐generational family history to screen for familial heart diseases. Also, all patients with no evidence of coronary disease underwent a cardiac magnetic resonance study to assess ventricular volumes and function, and for myocardial tissue characterization using late gadolinium enhancement to identify patterns suggestive of specific non‐ischaemic cardiomyopathies. In cases with a suspicion of a genetic cause, such as a positive family history or specific clinical features, multi‐gene panel genetic testing was performed in line with expert consensus statements. Other conditions, such as congenital heart diseases, myocarditis, left ventricular noncompaction and Takotsubo were defined according to current established standards.

The study was carried out according to the Declaration of Helsinki. Informed consent was obtained from all patients during the index admission, when all patients were thoroughly informed of the observational nature of the study and were asked to sign a written consent if available to participate. The study was approved by the local ethics committee (Comitato Etico Regionale delle Marche, n°173/2022). The present manuscript was designed, conducted, and reported according to the STROBE initiative.

### Data collection

A comprehensive cardiac assessment, including a 12‐lead ECG and echocardiography, was prospectively conducted for all patients on admission, at discharge and every 6 months thereafter. Expert physicians were tasked with prospectively gathering data on patients' demographics, risk factors, medical history and treatment. Continuous 12‐lead ECG monitoring (Mortara Rangoni, Arezzo, Italy) was utilized to monitor heart rate and detect underlying arrhythmias during hospitalization. Blood samples for brain natriuretic peptide and troponin I were obtained upon admission and at discharge. Echocardiographic assessments were conducted using a monoplane ultrasound probe (M4S) of Epiq CVxi echocardiograph (Philips Ultrasound, Bothell, WA, USA). Digital loops were captured, recording a minimum of three consecutive beats and analyzed offline using dedicated software as previously described.^6^

To ensure the comparability of drug regimens, a treatment intensity score (TIS) was computed. Following previous reports, the daily dose recorded for each patient was divided by the maximum recommended daily dose to derive a proportional dose for that medication, termed intensity. Maximum recommended daily doses were determined based on European and American guidelines.

All patients had a complete dataset during hospitalization and were followed up in our out‐of‐hospital clinic until a final diagnosis was made, with a minimum of 1 year for all patients. After that, they could choose to continue their follow‐up in our clinic or be referred to another institution for routine visits and be contacted remotely every 6 months for endpoints adjudication (see below).

### Endpoints

The primary endpoint was the incidence of all‐cause mortality in the four groups.

The secondary endpoint was the incidence of unplanned cardiovascular hospitalization in the four groups. Unplanned cardiovascular hospitalizations were defined as any unplanned hospitalization longer than 12 h for one or more of the following reasons: acute coronary syndrome, unstable angina, HF recurrence, atrial or ventricular arrhythmia, valvular heart disease, infective endocarditis, myocarditis, pericarditis, aortic disease, pulmonary embolism, stroke/transient ischaemic attack (TIA), syncope, cardiovascular‐related urgent procedures and complications of such procedures.

Data on mortality were cross‐referenced with the regional mortality registry, and data on hospitalizations were collected from patient records and verified through the regional hospital discharge database.

### Statistical analysis

All continuous variables were checked for normality through the Kolmogorov–Smirnov test. Normally distributed variables were described by mean and standard deviation and compared by analysis of variance. Not normally distributed variables were described as median and 1st–3rd interquartile range (IQR) and compared by non‐parametric tests. Categorical variables were described as prevalences and compared by *χ* ^2^ test or Fisher exact test, as appropriate. ANOVA for repeated measures was used to test changes in clinical, laboratory and treatment parameters during hospitalization and follow‐up. Univariate‐derived associations were adjusted by common clinical risk factors.

Kaplan–Meier analysis was used to assess time free from primary and secondary endpoints. In order to reduce the weight of potential confounders and different baseline characteristics, a propensity score for the likelihood of having a TCM diagnosis was obtained by means of multiple logistic regression. The variables included in the score were: age, gender, body mass index, NYHA class at presentation, hypertension, diabetes, dyslipidaemia, chronic obstructive pulmonary disease, previous stroke or TIA, previous acute coronary syndrome, previous acute coronary revascularization, heart rate at presentation, creatinine at admission, BNP at admission and LVEF at admission. Matching was then performed on a two‐versus‐two basis (pure TCM vs. non‐ischaemic HF; impure TCM vs. ischaemic HF) on log‐transformed propensity score in a 1:1 fashion with a calliper of 0.1 to account for the different baseline characteristics between the four groups. A sensitivity Kaplan–Meier analysis on the propensity matched samples was then performed on primary and secondary endpoints.

Another set of sensitivity analyses was performed using two different cut‐offs for LVEF improvement to label the patients as a pure TCM. In the first analysis, an improvement of at least 15% (rather than 5%) was used. In the second analysis, only patients with complete LVEF recovery (i.e., ≥50%) were labelled as ‘pure TCM’. All patients not meeting the stricter cut‐off were reassigned to the ‘impure TCM’ group, considering idiopathic dilated cardiomyopathy as the most likely underlying structural heart disease.

Within the TCM groups (both pure and impure), patients were divided according to the clinical strategy adopted during the first admission into rhythm‐control (pharmacological or electrical cardioversion and rhythm control drug at discharge or catheter ablation) or rate‐control (rate‐controlling drugs with no attempt at restoration of sinus rhythm). A Kaplan–Meier analysis was used to assess time free from primary and secondary endpoints between rhythm‐control and rate‐control groups.

A similar propensity score as described above, but matched between rhythm‐control and rate‐control groups, was used to test the association between rhythm and rate control and primary and secondary outcomes.

SPSS 25.0 for Windows (SPSS Inc., Chicago, IL, USA) and R (R Foundation for Statistical Computing, Vienna, Austria) were used for statistical analysis. Values of *P* < 0.05 (two‐tailed) were considered statistically significant.

## Results

### Baseline characteristics

We consecutively enrolled 456 patients (304 males, median age 72.8 years) and divided them into the four groups previously described (*Table* 1). The median follow‐up was 3 years (IQR 1.5–5.1 years).

**Table 1:** General characteristics of the population at admission, also divided by subgroups.

	Total	Pure TCM	Impure TCM	Non‐ischaemic HF	Ischaemic HF	*P*
	(*n* = 456)	(*n* = 125)	(*n* = 44)	(*n* = 82)	(*n* = 205)	
Male gender, *n* (%)	304 (66.7%)	77 (61.6%)	31 (70.5%)	51 (62.2%)	145 (70.7%)	0.2625
Age, years	72.8 (64.4–79.7)	70.9 (60.9–76.8)	75.1 (70.5–82.4)	70.5 (56.2–79.2)	75.2 (65.7–80.8)	**0.0356**
BMI, kg/m^2^	27.6 ± 5.3	28.5 ± 5.3	27.6 ± 5.9	27.8 ± 5.8	26.9 ± 4.8	0.0707
Arterial hypertension, *n* (%)	308 (67.8%)	80 (64.0%)	28 (63.6%)	49 (59.8%)	151 (74.4%)	0.0805
Diabetes, *n* (%)	123 (27.1%)	18 (14.4%)	11 (25.0%)	20 (24.4%)	74 (36.5%)	**0.0002**
Dyslipidaemia, *n* (%)	214 (47.1%)	43 (34.4%)	26 (59.1%)	28 (34.1%)	117 (57.6%)	**<0.0001**
Active smoking, *n* (%)	67 (14.8%)	14 (11.2%)	5 (11.4%)	16 (19.5%)	32 (15.8%)	**0.0143**
CKD, *n* (%)	128 (28.2%)	23 (18.5%)	14 (31.8%)	16 (19.5%)	75 (36.8%)	**0.0034**
COPD, *n* (%)	87 (19.1%)	16 (12.8%)	6 (13.6%)	10 (12.2%)	55 (27.0%)	**0.0020**
OSAS, *n* (%)	19 (4.2%)	7 (5.6%)	1 (2.3%)	4 (4.9%)	7 (3.4%)	0.7026
Previous stroke, *n* (%)	41 (9.0%)	4 (3.2%)	9 (20.5%)	3 (3.7%)	25 (12.3%)	**0.0015**
Previous MI, *n* (%)	80 (17.6%)	0 (0.0%)	8 (18.2%)	0 (0.0%)	72 (35.3%)	**0.0005**
Previous CABG, *n* (%)	26 (5.7%)	0 (0.0%)	3 (6.8%)	0 (0.0%)	23 (11.3%)	**0.0005**
Previous PCI, *n* (%)	49 (10.8%)	0 (0.0%)	5 (11.4%)	0 (0.0%)	44 (21.6%)	**0.0005**

Abbreviations: BMI, body mass index; CABG, coronary artery bypass graft; CKD, chronic kidney disease; COPD, chronic obstructive pulmonary disease; HF, heart failure; MI, myocardial infarction; OSAS, obstructive sleep apnoea syndrome; PCI, primary coronary intervention; TCM, tachycardia‐induced cardiomyopathy.

**Figure 2:**
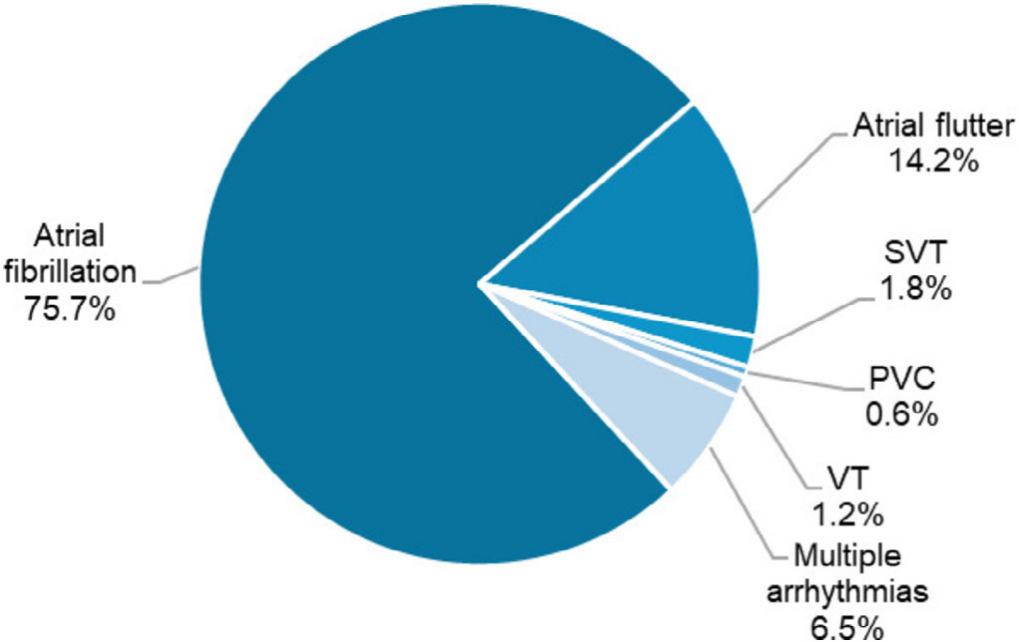
Culprit arrhythmias on admission in patients with tachycardia‐induced cardiomyopathy. PVC, premature ventricular contraction; SVT, supraventricular tachycardia; VT, ventricular tachycardia.

TCM was mainly due to AF (128 patients, 75.7%). *Figure* 2 details the prevalence of all types of arrhythmias on admission in patients with TCM (both pure and impure). A rhythm control strategy was chosen to treat the acute TCM phase in 124 patients (73.4%): Electric cardioversion was performed in 60.9% of all TCM, 8.4% underwent pharmacological cardioversion and 30.7% went straight to ablation during the first hospitalization. A rate control strategy was followed until discharge in the remaining 45 patients (26.6%).

Regarding patients classified as ischaemic HF, 43 (21.0%) underwent percutaneous coronary intervention, and 16 (7.8%) underwent coronary artery bypass graft for a concomitant diagnosis of acute coronary syndrome.

### Hospitalization and follow‐up

All four subgroups experienced changes in clinical and laboratory parameters during hospitalization (*Table* S1), with a significant improvement of NYHA class before discharge and a concomitant reduction of BNP. On a note, heart rate decreased in all four subgroups to a similar level right before discharge, although pure and impure TCM presented a higher heart rate on admission (119 ± 29 bpm vs. 85 ± 27 bpm in non‐TCM patients; *P* < 0.001).

TISs for all major HF drug classes, also divided by subgroups, are shown in *Table* S2.

**Figure 3:**
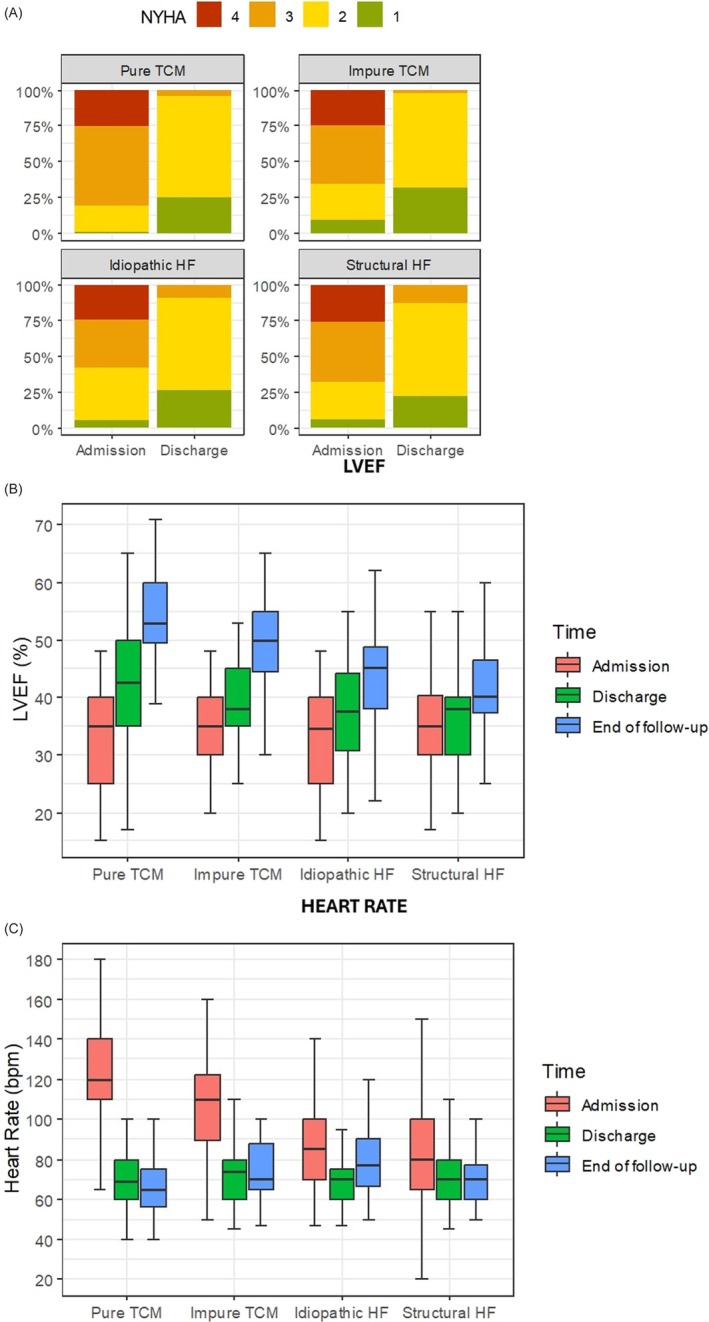
Changes in NYHA class (A), LVEF (B) and heart rate (C) throughout the study according to the different subgroups. HF, heart failure; LVEF, left ventricular ejection fraction; NYHA, New York Heart Association; TCM, tachycardia‐induced cardiomyopathy.

*Figure* 3 details the modifications in NYHA class (Panel A), LVEF (Panel B) and heart rate (Panel C) throughout the study according to the different subgroups.

### Primary endpoint

**Figure 4:**
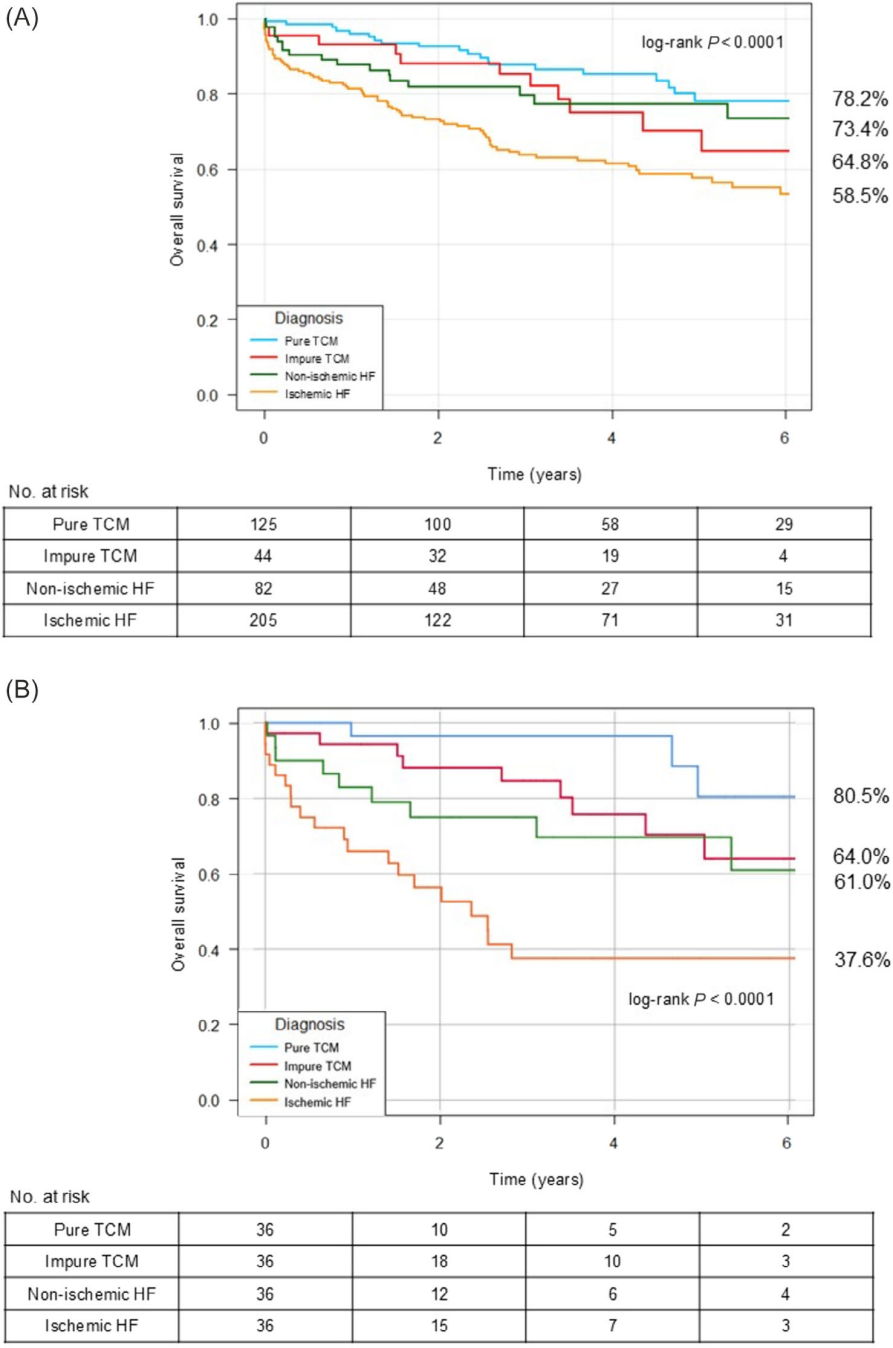
Kaplan–Meier curve: overall survival in the whole population (A) and in the propensity score‐matched population (B). HF, heart failure; TCM, tachycardia‐induced cardiomyopathy.

The four groups had significantly different estimates for all‐cause death, with pure TCM having the highest survival rate and ischaemic HF having the lowest survival rate over the follow‐up (pure TCM 78.2%; impure TCM 64.8%; non‐ischaemic HF 73.4%; ischaemic HF 58.5%; log‐rank *P* < 0.0001; *Figure* 4A). Using ischaemic HF as a comparator, HRs for death were significantly lower for patients with pure TCM [hazard ratio (HR) 0.34; 95% confidence interval (CI) 0.21–0.56] and non‐ischaemic HF (HR 0.53; 95% CI 0.32–0.90), while impure TCM did not differ (HR 0.57; 95% CI 0.30–1.07).

The propensity‐score matched analysis confirmed the results of the main Kaplan–Meier analysis (pure TCM 80.5%; impure TCM 64.0%; non‐ischaemic HF 61.0%; ischaemic HF 37.6%; log‐rank *P* < 0.0001; *Figure* 4B). In the propensity‐matched population, HRs for death were significantly lower for patients with pure TCM (HR 0.11; 95% CI 0.03–0.36), non‐ischaemic HF (HR 0.30; 95% CI 0.13–0.65) and impure TCM (HR 0.41; 95% CI 0.18–1.89), when using ischaemic HF as a comparator.

The sensitivity analyses using stricter cut‐offs for pure TCM confirmed the overall results of the primary analysis. No patients were reclassified when using an improvement of at least 15% of LVEF (i.e., all patients who improved more than 5% improved at least more than 15% during follow‐up), while using complete recovery (≥50%) as the cut‐off reassigned eight patients from pure to impure TCM groups. Nonetheless, even in the latter case, pure TCM had the highest survival rate, and ischaemic HF had the lowest survival rate over the follow‐up (pure TCM 76.9%; impure TCM 69.3%; non‐ischaemic HF 73.4%; ischaemic HF 58.5%; log‐rank *P* < 0.0001; *Figure* S2).

### Secondary endpoints

**Figure 5:**
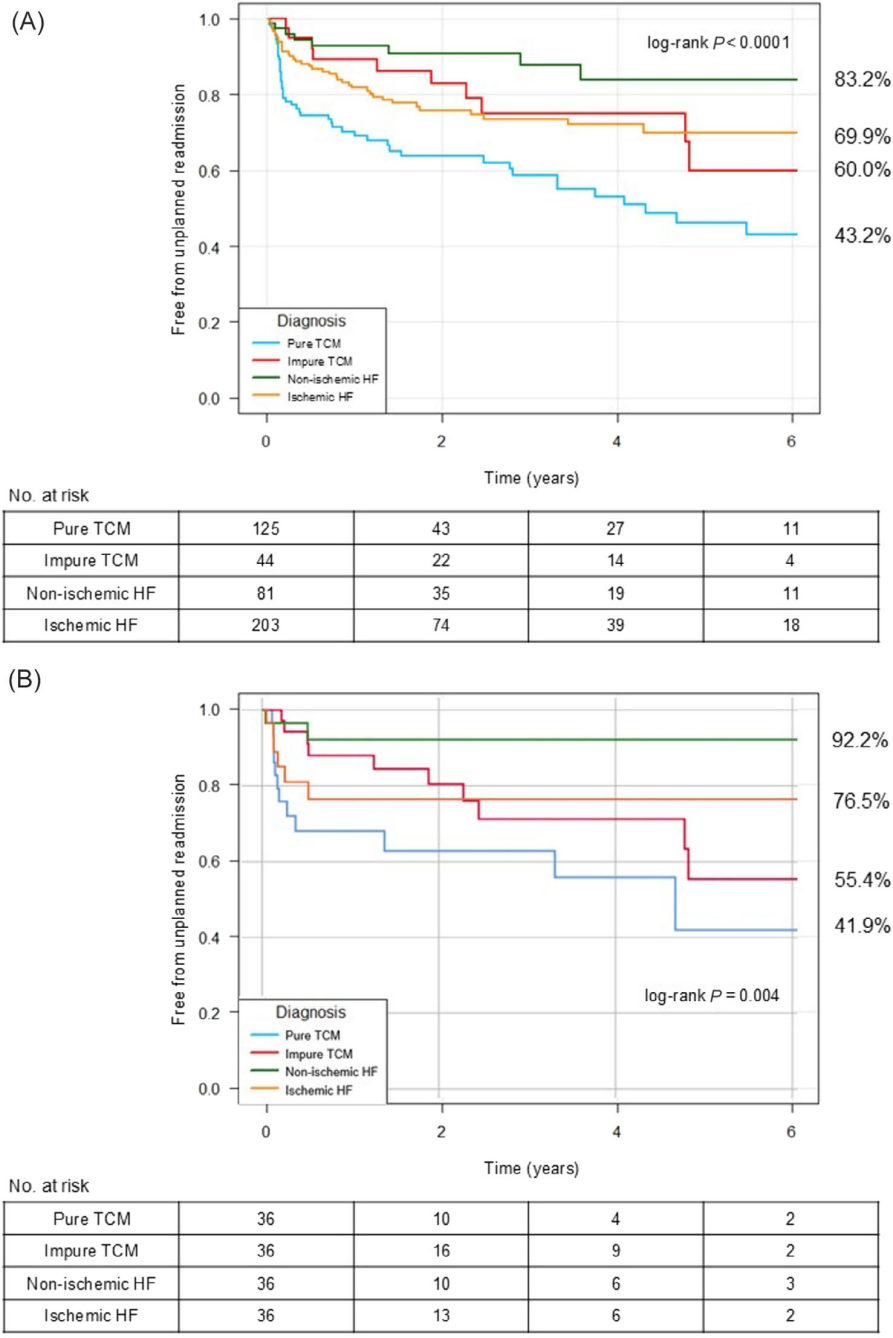
Kaplan–Meier curve: free‐from‐unplanned‐readmission estimates in the whole population (A) and in the propensity score‐matched population (B). HF, heart failure; TCM, tachycardia‐induced cardiomyopathy.

Unplanned hospitalizations showed a different trend compared with all‐cause mortality, with pure and impure TCM having the lowest free‐from‐readmission estimates over follow‐up (pure TCM 43.2%; impure TCM 60.0%; non‐ischaemic HF 83.2%; ischaemic HF 69.9%; log‐rank *P* < 0.0001; *Figure* 5A). Moreover, the average total number of unplanned hospitalizations also differed significantly between the four groups (pure TCM 0.81; 95% CI 0.62–1.00; impure TCM 0.84; 95% CI 0.51–1.17; non‐ischaemic HF 0.38; 95% CI 0.20–0.55; ischaemic HF 0.89; 95% CI 0.68–1.10; *P* = 0.019).

Unplanned hospitalizations for HF worsening were higher in the impure TCM group (22.7%) and lower in the other three groups (pure TCM 8.0%; non‐ischaemic HF 12.1%; ischaemic HF 18.0%; log‐rank *P* < 0.0001).

The sensitivity analysis using the propensity‐score matched groups confirmed the results on the larger population, with pure and impure TCM having the lowest free‐from‐readmission estimates over follow‐up (pure TCM 41.9%; impure TCM 55.4%; non‐ischaemic HF 92.2%; ischaemic HF 76.5%; log‐rank *P* = 0.004; *Figure* 5B).

Even for the secondary endpoint, using complete recovery as a strict cut‐off for pure TCM confirmed the overall results of the primary analysis, with pure and impure TCM having the lowest free‐from‐readmission estimates over follow‐up (pure TCM 45.4%; impure TCM 52.7%; non‐ischaemic HF 83.2%; ischaemic HF 69.9%; log‐rank *P* = 0.001; *Figure* S3).

### Outcomes in TCM: Rhythm versus rate control

Within the two TCM groups (*n* = 169) candidates for an initial rate control strategy were overall more frequently female, older and with a higher prevalence of hypertension and previous stroke (*Table* S3). During the index hospitalization, 30.7% of all TCM patients undergoing rhythm control had a catheter ablation scheduled and performed, while the others were discharged on pharmacological rhythm control‐only.

Within the TCM groups, an initial rhythm control strategy was associated with better overall survival (79.0% vs. 63.1%; log‐rank *P* < 0.0001; *Figure* S1A). The same analysis on the propensity‐matched population confirmed similar results (78.9% vs. 57.5%; log‐rank *P* = 0.037; *Figure* S1B).

The incidence of total unplanned hospitalizations did not differ between an initial rhythm or rate control strategy, with the former most frequently associated with AF recurrence (30% vs. 0%) and the latter associated with a higher risk of unplanned readmission for HF or TCM recurrence (57% vs. 21%). A subsequent analysis considering different strategies of rhythm control (pharmacological vs. catheter ablation) was deemed unfeasible due to the low sample size.

## Discussion

The present study presents some original, real‐world findings regarding the prognosis of different types of HFrEF and therefore may help guide their treatment and follow‐up.

Our data underlined distinct differences in survival and hospitalization rates between TCM subtypes (pure and impure) and other forms of HF. Pure TCM patients showed higher survival rates (*Figure* 4), aligning with previous research suggesting a reversible component in TCM once arrhythmias are managed.^11^, ^12^ Conversely, ischaemic HF had the poorest survival outcomes, reflecting this group's intrinsic structural dysfunction. This is also confirmed by the larger increase in LVEF seen in TCM during follow‐up (*Figure* 3B), despite a similar background HF therapy between the four groups. Therefore, the importance of classifying HF subtypes accurately from the first presentation is paramount for prognosis, management planning, and follow‐up.

Given the reversibility of LV dysfunction in TCM, particularly in the pure form, early recognition and prompt intervention are critical. Our results suggest that TCM should be considered as a first‐line working diagnosis in every de novo HF presentation associated with cardiac arrhythmia (especially AF). Conversely, rhythm control options, including catheter ablation, should be discussed early.^13^ Considering the recurring nature of TCM, a long‐term follow‐up protocol could benefit this patient group by promptly addressing recurrent arrhythmias and preventing further HF decompensations.^14^ As demonstrated before, clinical predictors, such as obstructive sleep apnoea syndrome, BNP and heart rate at discharge, represent reliable clinical predictors of TCM recurrence and should be considered.^6^

Our dataset underlines a contemporary approach to TCM, with a large predominance (~80%) of rhythm control as the preferred strategy for acute management. More importantly, patients who received rhythm control had significantly higher survival rates compared with those managed with rate control, indicating that rhythm control may be beneficial in TCM.^14^, ^15^ This is in line with the evidence linking early rhythm control as associated with a lower risk of adverse cardiovascular events, which could be especially important when AF is associated with acute HF.^14^, ^16^ Our data confirm the benefits of an early rhythm control strategy in the TCM sub‐setting, with early rhythm control associated with better overall survival and lower risk of unplanned readmission for HF worsening (both TCM and non‐TCM‐related). However, no significant difference was observed between rhythm and rate control in terms of reducing the total number of unplanned hospitalizations, with emergency consultations for AF or atrial flutter management much more frequent in patients undergoing rhythm control. These data might suggest that, while rhythm control could improve survival, it may not completely mitigate the risk of recurrent hospitalizations, and its impact on quality of life is unclear, particularly in patients with impure TCM. Moreover, a less lenient approach to rate control could prove useful due to the known importance of heart rate as a predictor of TCM recurrence.

Unplanned hospitalizations were notably higher in both TCM groups than in non‐ischaemic HF (*Figure* 5), especially among impure TCM patients, who had also higher rates of HF worsening. Moreover, the higher admission heart rates in TCM patients highlight the potentially significant hemodynamic impact of tachyarrhythmias (*Figure* 3C). This points to the complex management of patients with both HF and arrhythmias, when a heart team including both electrophysiologists and HF specialists might be required to provide combined treatments and a more comprehensive follow‐up. Moreover, as AF is by far the predominant arrhythmia in TCM patients, its detection is key and should be pursued in all patients presenting with new HF symptoms.

On the other hand, AF and HF are deeply intertwined in a bidirectional fashion. In this setting, the more recent pharmacological options for HF, such as the angiotensin receptor–neprilysin inhibitors or the SGLT2 inhibitors, have shown promising results in improving the positive remodelling of the left ventricle and reducing the ventricular arrhythmic burden in HF patients^17^, ^18^ and should not be discontinued even after improvement in EF.^19^ It remains unclear whether these positive effects could be translated to the atrial chambers, effectively providing a first‐line treatment for atrial arrhythmias and maybe preventing atrial cardiomyopathy.

## Limitations

Despite valuable insights, this study's findings are limited by the observational nature of the study and its single‐centre design, which may affect generalizability. Moreover, the enrolment was performed in a primary referral centre for arrhythmic disorders. Therefore, the prevalence of TCM (both pure and impure) could have been overestimated and is probably lower in a more generalist setting, even after taking into account the exclusion of cardiomyopathies, congenital heart diseases and myocarditis. Moreover, despite the propensity score matching, which greatly helped to balance covariates, reducing confounding and making the four groups more comparable, it discarded a significant portion of the sample, reducing statistical power and generalizability, and did not account for unmeasured confounders.

In the absence of a standard, universally agreed‐upon definition of TCM, we proposed criteria for TCM definition that might seem lenient and based on clinical and echocardiographic improvement after ruling out other structural heart diseases. While a stricter threshold, such as LVEF improvement ≥15% or a final complete LVEF recovery,^9^ could have been more specific, they provided very similar results in our sensitivity analyses, with all patients labelled as ‘pure TCM’ experiencing at least a 15% improvement in LVEF and only eight of them not reaching complete recovery. Moreover, using a stricter cut‐off for excluding an underlying idiopathic dilated or non‐dilated cardiomyopathy did not significantly change the results for both primary and secondary endpoints (*Figure*s S2 and S3), leaving the search for a widely accepted cut‐off still open.

Finally, while the study identifies trends in survival and unplanned hospitalization, it does not assess quality‐of‐life outcomes, especially regarding the necessity of planned intervention for rhythm control in TCM patients and planned procedures, such as coronary angiographies or device implantations. Future multicentre studies could further explore the long‐term impact of rhythm control on both survival and quality of life and evaluate the efficacy of combining rhythm control with treatments aimed at underlying structural disease in impure TCM.

## Conclusions

While TCM can be considered an overall ‘benign’ condition, especially when compared with other causes of HF, it is however burdened by a high rate of recurrence and unplanned hospitalization, which in turn could negatively impact long‐term quality of life. Early recognition of arrhythmias as a culprit for HF is paramount to adopt swift rhythm control strategies and a tailored follow‐up.

## Conflict of interest statement

All authors declare no conflicts of interest.

## Funding

This manuscript was supported by the Marche Polytechnic University (FFARB 2017).

## Supporting information


**Figure S1.** Kaplan–Meier curve: cumulative survival in the rhythm versus rate control subgroups (panel A) and in the propensity score‐matched population (panel B).

**Figure S1.** Supporting Information.

**Figure S2.** Kaplan–Meier curve: overall survival in the whole population using complete left ventricular ejection fraction recovery (≥50%) as cut‐off for pure tachycardiomyopathy.

**Figure S3.** Kaplan–Meier curve: free‐from‐unplanned‐readmission estimates in the whole population in the whole population using complete left ventricular ejection fraction recovery (≥50%) as cut‐off for pure tachycardiomyopathy.

**Table S1.** Changes in clinical and laboratory parameters during hospitalization, divided by subgroups.

**Table S2.** Changes in treatment intensity score of the major heart failure drug classes, divided by subgroups.

**Table S3.** General characteristics of patients with tachycardiomyopathy, according to initial rhythm or rate control strategy.
