# Supplementary figures and images for: Long‐term prognosis of pure and impure tachycardiomyopathy
Source: ESC Heart Fail. 2025 Oct 9;12(6):4288–98. doi: 10.1002/ehf2.15444 (PMC12719866; doi:10.1002/ehf2.15444)

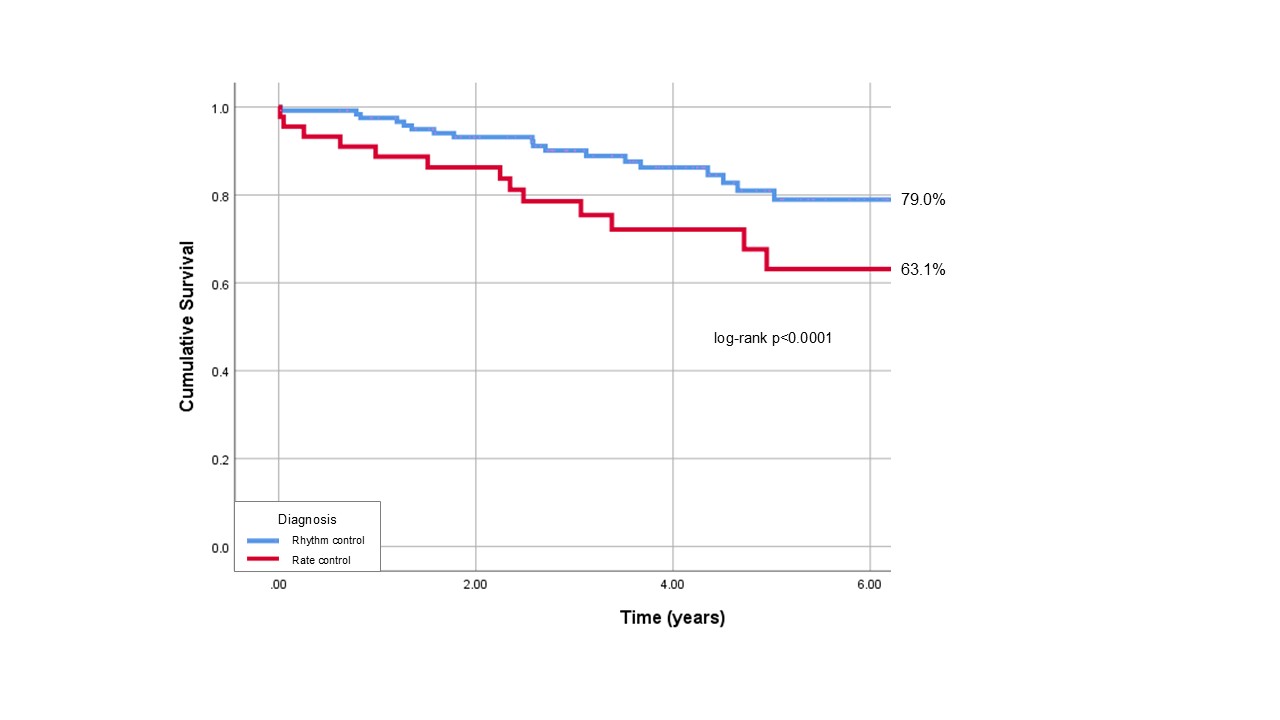

Supplement: Supplementary file 1 — Figure S1. Kaplan–Meier curve: cumulative survival in the rhythm versus rate control subgroups (panel A) and in the propensity score‐matched population (panel B). [file EHF2-12-4288-s002.jpg]

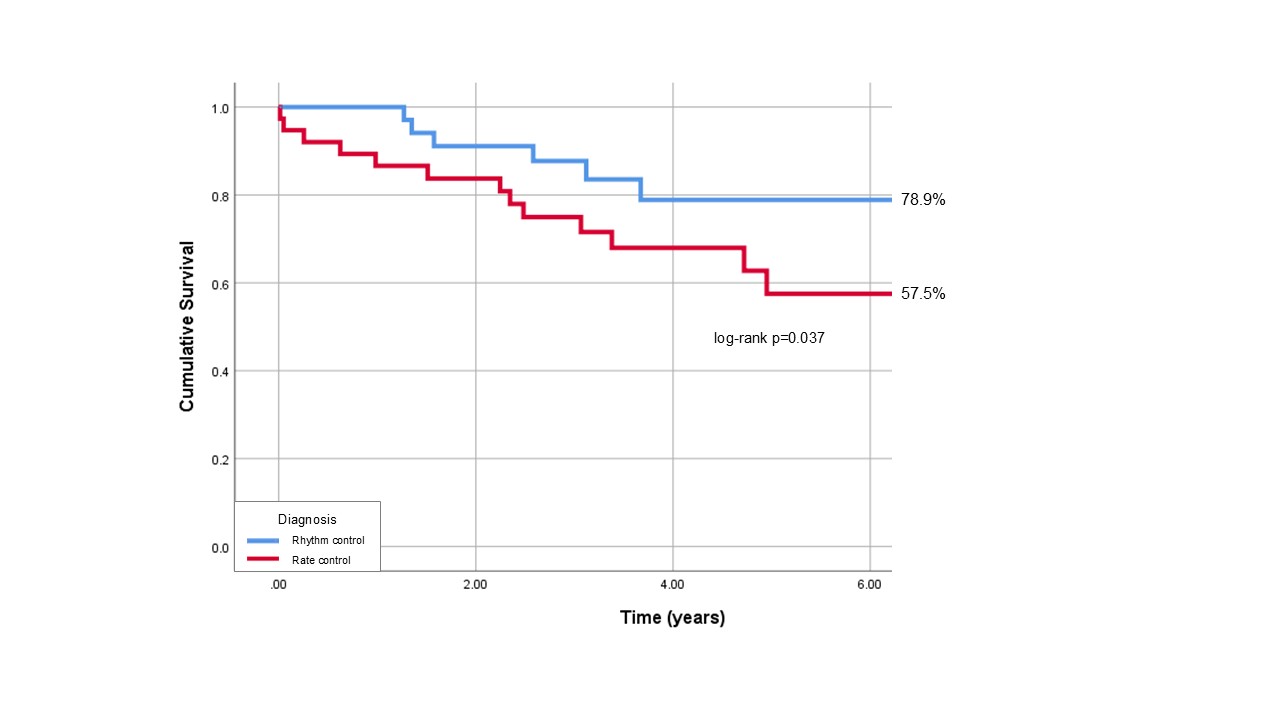

Supplement: Supplementary file 2 — Figure S1. Supporting Information. [file EHF2-12-4288-s005.jpg]

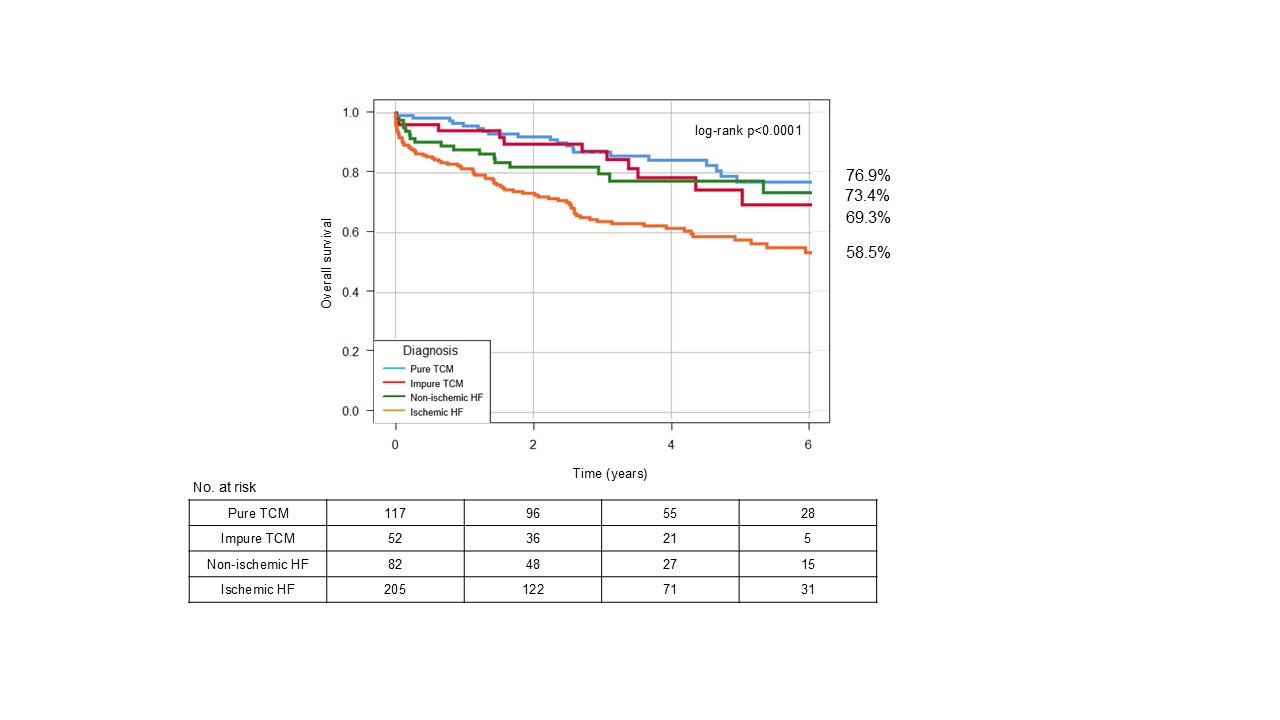

Supplement: Supplementary file 3 — Figure S2. Kaplan–Meier curve: overall survival in the whole population using complete left ventricular ejection fraction recovery (≥50%) as cut‐off for pure tachycardiomyopathy. [file EHF2-12-4288-s007.jpg]

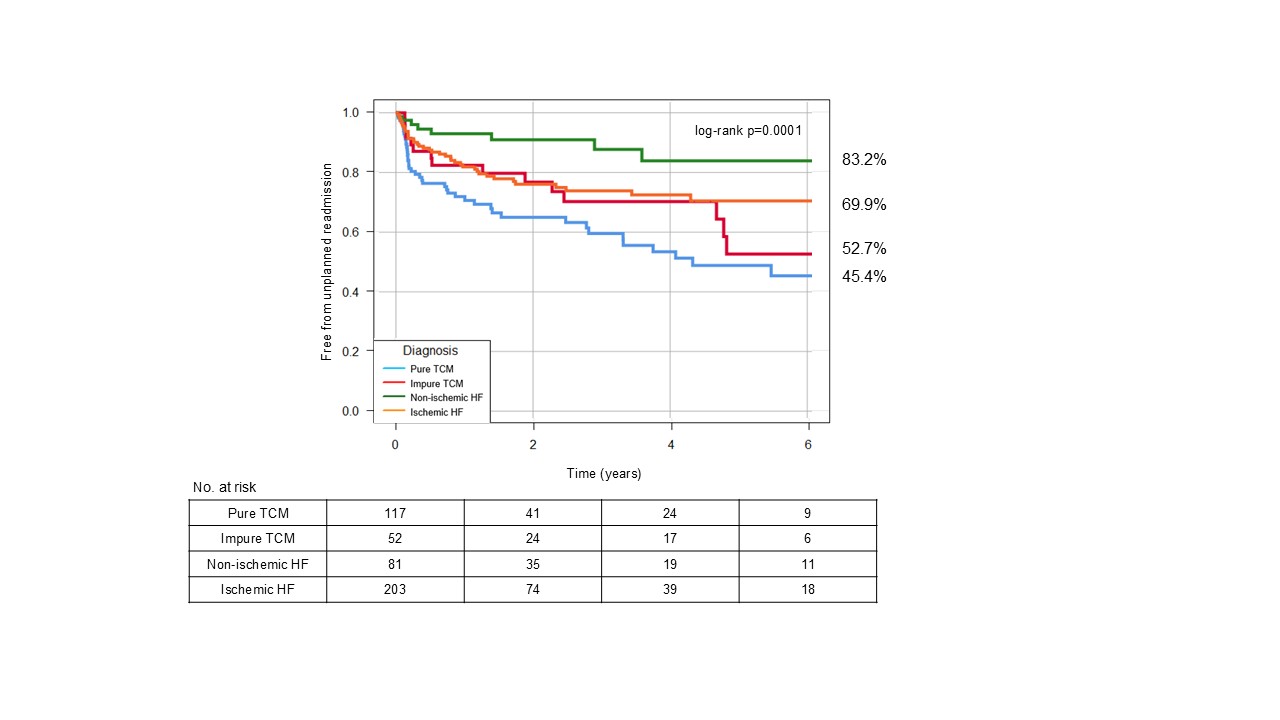

Supplement: Supplementary file 4 — Figure S3. Kaplan–Meier curve: free‐from‐unplanned‐readmission estimates in the whole population in the whole population using complete left ventricular ejection fraction recovery (≥50%) as cut‐off for pure tachycardiomyopathy. [file EHF2-12-4288-s004.jpg]
