# Supplementary material for: Long‐term prognosis of pure and impure tachycardiomyopathy
Source: ESC Heart Fail. 2025 Oct 9;12(6):4288–98. doi: 10.1002/ehf2.15444 (PMC12719866; doi:10.1002/ehf2.15444)
Supplement: Supplementary file 5 — Table S1. Changes in clinical and laboratory parameters during hospitalization, divided by subgroups. [file EHF2-12-4288-s006.docx]

**Supplementary Table 1.** **Changes in clinical and laboratory parameters during hospitalisation, divided by subgroups**

|  | **Pure TCM**  **(n=125)** | | | **Impure TCM**  **(n=44)** | | | **Non-ischemic HF**  **(n=82)** | | | **Ischemic HF**  **(n=205)** | | | |  |
| --- | --- | --- | --- | --- | --- | --- | --- | --- | --- | --- | --- | --- | --- | --- |
|  | **Admission** | **Discharge** | **p** | **Admission** | **Discharge** | **p** | **Admission** | **Discharge** | **p** | | **Admission** | **Discharge** | **p** | |
| NYHA class |  |  |  |  |  |  |  |  |  | |  |  |  | |
| II | 23 (18.5%) | 87 (70.7%) | **<0.001** | 11 (25.0%) | 29 (65.9%) | **<0.001** | 30 (36.6%) | 53 (64.6%) | **<0.001** | | 52 (25.5%) | 131 (65.8%) | **<0.001** | |
| III | 68 (54.8%) | 5 (4.1%) |  | 18 (40.9%) | 1 (2.3%) |  | 28 (34.1%) | 8 (9.8%) |  |  | 84 (41.2%) | 25 (12.6%) |  |  |
| IV | 32 (25.8%) | 0 (0.0%) |  | 11 (25.0%) | 0 (0.0%) |  | 20 (24.4%) | 0 (0.0%) |  |  | 54 (26.5%) | 0 (0.0%) |  |  |
| Heart rate (bpm) | 123.2±25.6 | 70.3±15.1 | **<0.001** | 107.9±29.2 | 72.6±15.3 | **<0.001** | 87.7±31.9 | 69.6±13.3 | **<0.001** | | 83.5±24.6 | 71.5±14.3 | **<0.001** | |
| Hemoglobin (g/l) | 13.5±2.0 | 13.4±2.0 | 0.777 | 13.1±2.3 | 12.6±2.0 | 0.105 | 13.0±2.2 | 12.8±2.5 | 0.546 | | 12.5±2.2 | 12.3±2.2 | 0.119 | |
| Creatinine (mg/dl) | 1.04(0.85-1.29) | 1.10(0.97-1.32) | **<0.001** | 1.18(0.90-1.40) | 1.32(1.08-1.60) | **0.003** | 1.08(0.89-1.44) | 1.26(1.10-1.44) | **0.018** | | 1.19(0.92-1.50) | 1.30(1.10-1.75) | **<0.001** | |
| Na+ (mEq/l) | 140.8±3.1 | 140.2±3.0 | 0.176 | 139.6±3.8 | 138.4±2.7 | 0.088 | 140.1±2.7 | 140.0±3.2 | 0.792 | | 139.7±4.1 | 139.9±3.5 | 0.692 | |
| K+ (mEq/l) | 4.26±0.48 | 4.19±0.43 | 0.239 | 4.19±0.53 | 4.24±0.37 | 0.739 | 4.18±0.56 | 4.19±0.53 | 0.887 | | 4.23±0.56 | 4.17±0.44 | 0.198 | |
| Troponin I (ng/l) | 0.58(0.07-1.59) | 0.07(0.02-1.66) | 0.419 | 0.04(0.02-8.75) | 0.03(0.02-0.08) | 0.205 | 11.00(0.10-42.75) | 0.25(0.08-14.80) | **0.033** | | 8.00(0.10-63.00) | 0.27(0.08-2.19) | **0.003** | |
| BNP (pg/ml) | 528(364-781) | 315(185-563) | **0.009** | 1020(645-1388) | 472(419-583) | **0.007** | 897(371-1195) | 187(141-564) | 0.092 | | 663(440-1126) | 409(166-794) | **0.003** | |
