# Supplementary material for: Long‐term prognosis of pure and impure tachycardiomyopathy
Source: ESC Heart Fail. 2025 Oct 9;12(6):4288–98. doi: 10.1002/ehf2.15444 (PMC12719866; doi:10.1002/ehf2.15444)
Supplement: Supplementary file 6 — Table S2. Changes in treatment intensity score of the major heart failure drug classes, divided by subgroups. [file EHF2-12-4288-s001.docx]

**Supplementary Table 2.** **Changes in treatment intensity score of the major heart failure drug classes, divided by subgroups**

|  | **Pure TCM**  **(n=125)** | | | **Impure TCM**  **(n=44)** | | | **Non-ischemic HF**  **(n=82)** | | | **Ischemic HF**  **(n=205)** | | |
| --- | --- | --- | --- | --- | --- | --- | --- | --- | --- | --- | --- | --- |
|  | **Discharge** | **End of FU** | **p** | **Discharge** | **End of FU** | **p** | **Discharge** | **End of FU** | **p** | **Discharge** | **End of FU** | **p** |
| ACE-I/ARB/ARNI | 0.34 (0.37-0.40) | 0.39 (0.33-0.44) | 0.164 | 0.27 (0.15-0.38) | 0.35 (0.24-0.46) | .265 | 0.22 (0.14-0.29) | 0.43 (0.35-0.50) | <0.001 | 0.24 (0.19-0.29) | 0.40 (0.35-0.45) | <0.001 |
| SGLT2-i | - | 0.76 (0.72-0.81) |  | - | 0.69 (0.64-0.73) |  | - | 0.68 (0.64-0.72) | - | - | 0.68 (0.65-0.71) | - |
| Beta-blocker | 0.20 (0.15-0.25) | 0.48 (0.43-0.53) | <0.001 | 0.17 (0.09-0.26) | 0.55 (0.47-0.64) | <0.001 | 0.22 (0.15-0.28) | 0.35 (0.30-0.41) | 0.003 | 0.22 (0.17-0.26) | 0.40 (0.35-0.44) | <0.001 |
| MRA | 0.12 (0.07-0.16) | 0.39 (0.33-0.43) | <0.001 | 0.09 (0.03-0.14) | 0.44 (0.34-0.55) | <0.001 | 0.10 (0.04-0.16) | 0.43 (0.36-0.51) | <0.001 | 0.10 (0.07-0.14) | 0.40 (0.34-0.45) | <0.001 |
| Ivabradine | 0 (0-0) | 0.01 (0.01-0.03) | 0.115 | 0.02 (0.01-0.04) | 0.04 (0.01-0.09) | 0.197 | 0.04 (0.01-0.08) | 0.12 (0.05-0.18) | 0.018 | 0.04 (0.01-0.06) | 0.08 (0.05-0.11) | 0.029 |
| Digoxin | 0.06 (0.02-0.10) | 0.04 (0.02-0.07 | 0.488 | 0.05 (0.01-0.12) | 0.05 (0.01-0.10) | 0.994 | 0.01 (0.01-0.02) | 0.07 (0.01-0.12) | 0.276 | 0.03 (0.01-0.05) | 0.05 (0.03-0.07) | 0.098 |
| Loop diuretic | 42 (35-48) | 21 (15-27) | <0.001 | 56 (46-67) | 18 (8-28) | <0.001 | 43 (36-51) | 18 (10-27) | <0.001 | 58 (53-64) | 30 (23-36) | <0.001 |

ACE-I: angiotensin converting enzyme-inhibitor; ARB: angiotensin receptor blocker; MRA: mineralocorticoid receptor antagonist; SGLT2-I: Sodium-glucose cotransporter-2 inhibitor. Note: for loop diuretics, the total daily dose was reported instead (in mg), as there is no recommended maximum dose to base a treatment intensity score upon. No SGLT2-I data were available at discharge as these drugs were approved for HF patients in Italy after the end of the enrolment (January 2022).
