# Supplementary material for: Long‐term prognosis of pure and impure tachycardiomyopathy
Source: ESC Heart Fail. 2025 Oct 9;12(6):4288–98. doi: 10.1002/ehf2.15444 (PMC12719866; doi:10.1002/ehf2.15444)
Supplement: Supplementary file 7 — Table S3. General characteristics of patients with tachycardiomyopathy, according to initial rhythm or rate control strategy. [file EHF2-12-4288-s003.docx]

**Supplementary Table 3. General characteristics of patients with tachycardiomyopathy, according to initial rhythm or rate control strategy**

|  | **Total**  **(n=169)** | **Rhythm control**  **(n=124)** | **Rate control**  **(n=45)** | **p** |
| --- | --- | --- | --- | --- |
| Male gender (n, %) | 108 (63.9%) | 85 (68.5%) | 23 (51.1%) | **0.0372** |
| Age (years) | 71.8 (64.7-78.7) | 68.3 (60.1-76.1) | 75.3 (68.9-83.8) | **0.0046** |
| BMI (kg/m^2^) | 28.2±5.3 | 28.4±4.8 | 27.9±7.1 | 0.5859 |
| Arterial hypertension (n, %) | 108 (63.9%) | 73 (58.9%) | 35 (77.8%) | **0.0236** |
| Diabetes (n, %) | 29 (17.2%) | 18 (14.5%) | 11 (24.4%) | 0.1304 |
| Dyslipidaemia (n, %) | 69 (40.8%) | 49 (39.5%) | 20 (44.4%) | 0.5651 |
| Active smoking (n, %) | 19 (11.2%) | 13 (10.5%) | 6 (13.3%) | 0.3648 |
| CKD (n, %) | 37 (22.0%) | 24 (19.5%) | 13 (28.9%) | 0.1943 |
| COPD (n, %) | 22 (13.0%) | 15 (12.1%) | 7 (15.6%) | 0.5553 |
| OSAS (n, %) | 8 (4.8%) | 6 (4.8%) | 2 (4.5%) | 0.9369 |
| Previous stroke (n, %) | 13 (7.7%) | 5 (4.0%) | 8 (17.8%) | **0.0063** |
| Previous MI (n, %) | 8 (4.7%) | 5 (4.0%) | 3 (6.7%) | 0.4762 |
| Previous CABG (n, %) | 3 (1.8%) | 1 (0.8%) | 2 (4.4%) | 0.1133 |
| Previous PCI (n, %) | 5 (3.0%) | 3 (2.4%) | 2 (4.4%) | 0.4919 |
| *Initial anti-arrhythmic strategy:* |  |  |  |  |
| Pharmacological rhythm control (n, %) | 86 (50.9%) | 86 (69.3%) | 0 (0%) | - |
| Catheter ablation (n, %) | 38 (22.5%) | 38 (30.7%) | 0 (0%) | - |
| Pharmacological rate control (n, %) | 45 (26.6%) | 0 (0%) | 45 (100%) | - |
| NYHA class |  |  |  | 0.5768 |
| II | 34 (20.2%) | 24 (19.4%) | 10 (22.7%) |  |
| III | 86 (51.2%) | 63 (50.8%) | 53 (52.3%) |  |
| IV | 43 (25.6%) | 32 (25.8%) | 11 (25.0%) |  |
| Heart rate (bpm) | 119.6±29.7 | 120.7±29.0 | 116.7±31.6 | 0.4495 |
| Hemoglobin (g/l) | 13.5±2.0 | 13.9±1.7 | 12.5±2.1 | **0.0003** |
| Creatinine (mg/dl) | 1.12 (0.89-1.30) | 1.09 (0.89-1.33) | 1.21 (0.90-1.30) | 0.3026 |
| Na+ (mEq/l) | 140.3±3.2 | 140.6±2.8 | 139.6±4.0 | 0.1215 |
| K+ (mEq/l) | 4.3±0.5 | 4.3±0.5 | 4.2±0.5 | 0.6020 |
| Troponin I (ng/l) | 0.46 (0.01-3.64) | 0.52 (0.02-3.00) | 0.39 (0.01-15.00) | 0.5077 |
| BNP (pg/ml) | 535 (271-788) | 505 (249-647) | 680 (413-1285) | **0.0084** |
| LVEF (%) | 33.5±8.6 | 32.9±8.8 | 35.6±7.6 | 0.0665 |

BMI: body mass index; BNP: brain natriuretic peptide; CABG: coronary artery bypass graft; CKD: chronic kidney disease; COPD: chronic obstructive pulmonary disease; LVEF: left ventricular ejection fraction; MI: myocardial infarction; OSAS: obstructive sleep apnea syndrome; PCI: primary coronary intervention
